# Cyberbullying in Social Media and Online Games among Chinese College Students and Its Associated Factors

**DOI:** 10.3390/ijerph18094819

**Published:** 2021-04-30

**Authors:** Jinyu Huang, Zhaohao Zhong, Haoyuan Zhang, Liping Li

**Affiliations:** 1 Injury Prevention Research Center, Shantou University Medical College, Shantou 515041, China; 19jyhuang@stu.edu.cn (J.H.); 20zhzhong@stu.edu.cn (Z.Z.); 2 School of Public Health, Shantou University, Shantou 515041, China; 3 Administrative Office, Public Utilities Bureau of Shenzhen Shenshan Special Cooperation Zone, Shenzhen 518200, China; 17hyzhang@alumni.stu.edu.cn

**Keywords:** cyberbullying, incidence, associated factors, college students

## Abstract

Cyberbullying can have a terrible impact on the physical and mental health of those involved. In severe cases, some of those involved develop anxiety, depression, and suicidal tendencies. However, few studies focus on cyberbullying among Chinese college students. We aimed to understand the incidence of cyberbullying in social media and online games and its associated factors among college students in China. A cross-sectional STAR questionnaire survey was conducted for college students from the end of June to the beginning of July 2019. Selected via the method of cluster random sampling, students graded 1–5 (college) from two colleges in Shantou were invited to participate in the survey. Information was collected regarding respondents’ socio-demographic information, cyberbullying in social media and online games, self-esteem, anxiety symptoms, Internet addiction, etc. A binary logistic regression model was employed to use all significant variables tested using χ² test or t-test for estimating the effect of potential factors on cyberbullying among college students. Participants were 20.43 ± 1.513 (X ± SD) years old, and the age range was 15 to 25 years old. 64.32% college students reported that they had suffered from cyberbullying, and 25.98% reported bullying others online during the semester. Gender, anxiety symptoms, Internet addiction, game time, and violent elements in games were associated with cyberbullying in social media and online games among college students in China. In conclusion, cyberbullying in social media and online games is prevalent among college students in China. The above data provided insights that targeted and effective measures should be taken to prevent college students from cyberbullying.

## 1. Introduction

With the development of the times, the Internet industry has made great progress and it has been successfully integrated into our daily life. Information and communications technology has had a huge impact on the way that people deal with their relationships. It can be likened to a double-edged sword. The most serious negative effect was cyberbullying, which means using the Internet to cause malicious, repetitive, and hostile harm to individuals or groups.

At present, there is not a unified definition of cyberbullying in the world. By means of previous research [1,2,3,4], we define cyberbullying as using electronic communication technology (computer, phone, tablet, etc.) to cause harm or hostility to others. It may include making fun of others, isolating, and spreading rumors about others.

We can communicate with each other through the Internet without being face-to-face. Although we do not meet in person, some people may have conflicting opinions or verbal conflicts. Then, they could vent their dissatisfaction, even abuse, harass and bully others through social media, text messaging, email, etc. With the popularity of the network, cyberbullying, an emerging behavior, is also becoming more serious. Recently, many scholars have been aware that cyberbullying can have a terrible impact on the physical and mental health of teenagers; it can lead to heartbreak, embarrassment, humiliation, and marginalization. Additionally, some teenagers intend to seek revenge [4]. In severe cases, some teenagers develop anxiety, depression, and suicidal tendencies [5,6]. Except for psychological damage, cyberbullying may have more terrible impacts. Although the network and reality are far apart, some of the more serious cases of cyberbullying can turn into actual violence when they think they cannot solve their conflicts on the Internet [7,8]. Studies have found that cyberbullying was positively correlated with actual bullying, which means that those who bullied others online were also more likely to bully others in real life, and vice versa [9,10].

Originally, most of the research subjects of cyberbullying were teenagers. However, few studies focused on cyberbullying among undergraduates [4]. Besides, most studies about the associated factors of cyberbullying did not make a distinction between different channels of cyberbullying.

As definitions about cyberbullying vary from study to study, rates of cyberbullying vary widely from previous research. Previous studies [9,11,12,13,14,15,16,17,18,19] showed that the incidence of cyberbullying victimization was 2.7–84.9% (victimization rates); the incidence of cyberbullying was 2.0–43.7% (cyberbullying rates). In addition, previous studies [20,21,22,23] have found that gender, self-esteem, anxiety and Internet addiction may be related to Internet bullying, to some extent. Self-esteem describes how people evaluate themselves and the extent to which they accept themselves [24]. Anxiety is a condition characterized by anxiousness, fear, distress, and perceived threats in the environment or internal to an individual [25]. Internet addiction refers to the uncontrollable, excessive, and compulsive use of the Internet [26].

Now, overseas research is also increasingly focused on cyberbullying in social media. The proportion of 18 to 29-year-olds using social media rose from 9.0% to 89.0% from 2004 to 2014 [5]. Meanwhile, online games have gradually become one of the most popular leisure activities among young people. It attracts so many players of all genders, ages, and races. However, there is a lot of hostility in online games, especially competitive games, which are full of abuse and harassment [27]. However, cyberbullying in online games was rarely studied.

Therefore, the purpose of this study was to understand the incidence of cyberbullying among college students in social media and online games and aimed to know the associated factors of college students implementing and suffering cyberbullying, which could be of great help for us to take targeted prevention and intervention measures to reduce the incidence of cyberbullying and its negative impact on college students.

**Hypothesis** **1** ** (H1).** *Chinese college students have a high incidence of cyberbullying in social media and online games*.

**Hypothesis** **2** ** (H2).** *Identify the potential risk factors of cyberbullying among Chinese college students in social media and online games*.

## 2. Methods

### 2.1. Study Participants and Procedure

The research data were powered by www.wjx.cn (accessed on 22 June 2019), a platform providing functions equivalent to Amazon Mechanical Turk. Selected via the method of cluster random sampling, students graded 1–5 (college) from two colleges in Shantou were invited to participate in the survey. From the end of June to the beginning of July 2019, QR (Quick Response) codes were distributed to college students in Shantou city, Guangdong province for a questionnaire survey. College teachers made situation statements and distributed online questionnaires. This questionnaire set the IP address answering limit and WeChat answering limit. All participating college students needed to use WeChat to answer, and the same IP and WeChat ID could only answer once. After completing the questionnaire and passing the review, they received a random reward (4–10 yuan). Participants were not aware of the rewards until they completed the questionnaire to ensure that the rewards did not affect the representativeness of the sample. Next, we reviewed and eliminated invalid questionnaires. The criteria were: (1) Answer time less than 4 min; (2) Repeated questions with different answers; (3) Reverse questions were set in the scale. If the students choose the same option for all the questions, for example, choose the first option for all the questions, they would also be judged as invalid.

Finally, 1140 questionnaires were received, and 897 were valid; the effective rate was 78.68%. Participants were 20.43 ± 1.513 (X ± SD) years old, and the age range was 15 to 25 years old. Among them, 393 were males, accounting for 43.81%; 504 were females, accounting for 56.19%.

### 2.2. Data Collection

The design of the questionnaire was based on previous research [28,29]. The questionnaires of this research included different ways of being bullied, such as text messages, photo/video messages, phone calls, etc. However, through the preliminary survey and interview, we found that it was rare for college students in Shantou to use e-mail, fixed phones and text messages to bully others or to be bullied. Forums, blogs, and chat rooms were also rare. Moreover, we did not find any cyberbullying behaviors such as stealthily photographing, stealing other people’s information, and pretending to be others’ identities. This was inconsistent with the results of many existing European and American studies [30]. Therefore, we cannot use foreign questionnaires directly. Based on previous research [31,32], pre-survey and interview, we identified that social media and online games were the most common places for cyberbullying. It was also in line with the current trend in the prevalence of cyberbullying [33]. After consulting a large amount of literature, we designed the cyberbullying questionnaire by ourselves. After a review by experts, the questionnaire was adjusted and modified. The content of the questionnaire includes respondents’ sociodemographic information, cyberbullying in social media and online games, self-esteem, anxiety symptoms, Internet addiction, etc.

We used Rosenberg’s [34] self-esteem scale (RSES) to evaluate the self-esteem of college students. The RSES includes 10 items, each of which contained four options, namely “very inconsistent”, “not consistent”, “consistent” and “very consistent”, with corresponding scores of 1, 2, 3, and 4, respectively. The total score of RSES ranged from 10 to 40, and higher scores indicated higher self-esteem. In this study, the Cronbach’s coefficient of RSES was 0.928, which means that the data were reliable. In addition, the validity analysis of RSES was also analyzed. KMO (Kaiser-Meyer-Olkin) value was 0.95, Bartlett’s spherical test’s Bart spherical value was 5629.08, *p* < 0.001, and the cumulative variance interpretation rate (after rotation) was 60.97%, indicating that RSES had a good validity.

W. K. Zung’s [35] self-anxiety scale (SAS) was used to evaluate the anxiety of college students. SAS includes 20 items, each of which contains 4 options, namely “no or very little time”, “sometimes”, “most of the time” and “most or all of the time”, with corresponding scores of 1, 2, 3 and 4, respectively. The total score of SAS is the rough score (X) obtained by adding the scores of 20 items. After formula conversion, the rough score multiplied by 1.25 is taken as the standard score. In this study, the Cronbach’s coefficient of SAS was 0.878. In the validity analysis, the KMO value was 0.938, the Bart spherical value was 7406.92, *p* < 0.001, and the cumulative variance interpretation rate (after rotation) was 53.66%, indicating that the reliability and validity of SAS were good.

Revised Chinese Internet Addiction Scale (CIAS-R) [36] (online Supplementary Table S1) was used to evaluate the Internet addiction status of college students. CIAS-R includes 19 items, each of which contains four options, namely “strongly inconsistent”, “inconsistent”, “consistent” and “very consistent”, with corresponding scores of 1, 2, 3, and 4, respectively. The CIAS-R score ranged from 19 to 76 points. In this study, the Cronbach’s coefficient of CIAS-R was 0.94, indicating that the data reliable quality was high. In addition, the validity analysis of CIAS-R was also analyzed, and the KMO value was 0.96; Bartlett’s spherical value was 8951.46, *p* < 0.001, and the cumulative variance interpretation rate (after rotation) was 54.11%, indicating that CIAS-R had good validity.

### 2.3. Ethics

This study was approved by the Ethics Committee of Shantou University Medical School (No. SUMC-2019-68) and we have also obtained the consent of the school’s student affairs office and counselors. All participants gave their informed consent and volunteered to participate.

### 2.4. Statistical Analysis

Descriptive statistics were performed to evaluate participant characteristics. Continuous and categorical variables were presented as mean (standard deviation, SD) and number (percentage), respectively, and tested for between-group differences by χ² test or *t*-test. A binary logistic regression model was employed to consider all significant variables tested using χ² test or t-test together for estimating the effect of associated factors on cyberbullying; the final model included odds ratios (ORs) and 95% confidence intervals (95% CIs). Statistical Product and Service Solutions 19.0 (SPSS, Inc., Chicago, IL, USA) was used for statistical analyses. SPSS has excellent functions, such as statistical analysis, chart analysis, data management, output management, which is one of the most commonly used statistical analysis software at present. Compared with other statistical software, although its programming function is not comprehensive, it has the following advantages. It has better functionality, with a complete editing, statistics, drawing, data input function. In addition, its programming is simple and convenient. Therefore, users who are familiar with the principle of statistical analysis can obtain the required results and are not prone to erroneous results [37]. A two-sided *p*-value < 0.05 was considered to be statistically significant.

## 3. Results

### 3.1. Incidence of Cyberbullying

Participants were 20.43 ± 1.513 (X ± SD) years old, and the age range was 15 to 25 years old. Based on the definition of cyberbullying given in this study, a total of 577 (64.32%) college students had suffered cyberbullying during the semester. Among them, the victimization rate of cyberbullying was 79.64% for males (313/393) and 52.38% for females (264/504). Overall, 461 college students (51.39%) had been bullied on social media, and 355 college students (39.58%) had been bullied on online games.

A total of 233 (25.98%) college students report bullying others online within this semester. The perpetration rate of cyberbullying was 42.49% for males (167/393) and 13.09% for females (66/504). Furthermore, 174 (19.40%) college students reported that they had bullied others on social media, and 122 (13.60%) college students reported that they had bullied others in online games.

### 3.2. Univariate Analysis of Cyberbullying in Social Media

We conducted a univariate analysis of cyberbullying in social media. A total of 11 factors were included, including gender, daily Internet time, college, grade, nationality, single-child family, native Cantonese, parents’ marital status, self-esteem, anxiety symptoms, and Internet addiction. Univariate analysis showed that the factors associated with being bullied on social media were gender (χ² = 52.099, *p* < 0.001), anxiety symptoms (t = 6.647, *p* < 0.001), and Internet addiction (t = 4.442, *p* < 0.001). Factors associated with bullying others in social media were gender (χ² = 50.525, *p* < 0.001), college (χ² = 6.983, *p* = 0.013), anxiety symptoms (t = 4.764, *p* < 0.001) and Internet addiction (t = 3.949, *p* < 0.001), as shown in Table 1 and Table 2.

### 3.3. Univariate Analysis of Cyberbullying in Online Games

We conducted a univariate analysis of cyberbullying in online games. A total of 12 factors were included, including gender, time spent on online games, college, grade, nationality, single-child family, native Cantonese, parents’ marital status, the game with violent elements (refers to the content of fighting, kidnapping, rape, murder and war terror in the game), self-esteem, anxiety symptoms, and Internet addiction. Univariate analysis showed that the factors associated with being bullied in online games were gender (χ² = 33.658, *p* < 0.001), game time per week (χ² = 37.996, *p* < 0.001), and game with violent elements (χ² =107.258, *p* < 0.001). Factors associated with bullying others in the online games were gender (χ² = 56.603, *p* < 0.001), game time per week (χ² =45.184, *p* < 0.001), and game with violent elements (χ² =40.730, *p* < 0.001), as shown in Table 3 and Table 4.

### 3.4. Multivariate Analysis of Cyberbullying in Social Media

All factors with statistically significant differences in the univariate analysis by χ² test and *t*-test were included in the final model for multivariate logistic regression analysis. We have found that factors associated with being bullied in social media were gender, anxiety symptoms, and Internet addiction. Male (OR = 3.192, 95%CI:2.389~4.264), higher scores of SAS (OR = 1.048, 95%CI: 1.030~1.066) and higher scores of CIAS-R (OR =1.025, 95%CI: 1.010~1.040) tended to have a higher risk of being bullied on social media.

Factors associated with bullying others on social media included gender, anxiety symptoms, and Internet addiction. Men (OR = 4.024, 95%CI:2.738~5.817), higher scores of SAS (OR = 1.044, 95%CI:1.024~1.063), and higher scores of CIAS-R (OR = 1.029, 95%CI:1.010~1.049) were more likely to bully others on social media.

### 3.5. Multivariate Analysis of Cyberbullying in Online Games

Factors related to being bullied in online games included gender, game time weekly, violent elements in games, and Internet addiction. Men (OR = 2.141, 95%CI: 1.437~3.192) were more likely to be bullied than women on online games. Students who played online games that were prone to conflict, such as violence and confrontation (OR = 5.668, 95%CI:3.911~8.214), were more likely to suffer from cyberbullying than those who played games that were not. Students who spent more time on online games (OR = 1.404, 95%CI:1.091~1.807) have a higher risk of being bullied; students with higher scores of CIAS-R (OR = 1.018, 95%CI:1.001~1.037) were more likely to suffer cyberbullying.

Factors related to bullying others in online games included gender, time spent on online games per week, and violent elements in games. Males were more likely to bully others in online games than females (OR = 4.676, 95%CI: 2.673–8.179). Students who played games with violence, confrontation, and other risks of conflict were more likely to bully others than those who do not play games without violence, confrontation, and other risks of conflict (OR = 3.969, 95%CI: 2.332–6.756). Students with a long playing time were more likely to bully others (OR = 1.461, 95%CI: 1.155~1.847), as shown in Table 5.

## 4. Discussion

### 4.1. Incidence of Cyberbullying

The results showed that the victimization rate of cyberbullying among college students in Shantou (including cyberbullying on social media and online games) was 64.32% (577 people), and the perpetration rate of cyberbullying was 25.98% (233 people). The results were higher than a Chinese study on college students’ cyberbullying in 2015 [38]. Their research report indicated that the victimization rate of college students’ cyberbullying was 39.18% and the perpetration rate was 17.32%. The reason for the different results of two studies may be the inconsistency of the definition of cyberbullying. In addition, the survey time set in this study was within this semester, but their time range was not mentioned. We paid much attention to research cyberbullying in online games and social media, but they only looked at social media. Last but not least, this study was conducted in 2019, and their study was conducted in 2015. According to the 35th Statistical Report on Internet Development in China published by China Internet Network Information Center (CNNIC) in 2015 [39], there are 649 million Internet users in China, with an Internet penetration rate of only 47.9%. However, the 44th Statistical Report on The Development of The Internet in China published by CNNIC in 2019 [40] pointed out that by June 2019, the number of Chinese netizens had reached 854 million, with an Internet penetration rate of 61.2%. All of the above reasons could lead to differences between perpetration rate and victimization rate in different studies of cyberbullying. Our study results showed that cyberbullying in social media and online games was prevalent among college students in China. At the same time, it also showed that the incidence of cyberbullying among Chinese college students has increased significantly. Therefore, some measures should be taken to prevent college students from cyberbullying. Relevant departments should strengthen the network moral education of college students and ensure that college students can timely report to relevant departments and seek help when others or themselves encounter cyberbullying. In addition, the colleges should increase the content of network security education in relevant courses to improve the network security awareness of college students.

### 4.2. Factors Associated with Cyberbullying

Multivariate logical regression was the most common statistical analysis method to analyze associated factors. It has the advantages of exploring the influence of multiple factors on a certain factor and strong interpretability of the model [41,42]. Multivariate logistic regression results showed that men were both risk factors of being bullied, and bullying others on social media, which was consistent with many studies [20]. A possible reason was that men were more aggressive and impulsive than women [43,44]. Daily time spent on Internet had nothing to do with social media network bullying, probably because of the daily time on the Internet, which included social media time, learning, entertainment, information consulting, etc. It means that students who spend more time online do not necessarily spend more time on social media. Spending more daily time on social media could be related to social media bullying [20]. Cyberbullying had nothing to do with the type of college, which might be related to the small sample size of students from other colleges, and a more balanced sample should be selected for future research. Anxiety symptoms were a risk factor of being cyberbullied and bullying others. Some researchers proposed that anxiety was an important predictor and outcome of bullying involvement [21,45], which was similar to this study. Besides, Internet addiction was a risk factor of being bullied and bullying others. Several possibilities might be attributed to this phenomenon. Firstly, intensive use of the internet was associated with the likelihood of cyberbullying [22]. Secondly, based on the cognitive-behavioral model of Davis [46], Internet addiction was conceptualized as “an impulse control disorder” and found to be related to a wide range of psychosocial complications, including cyberbullying [47].

In online games, being a man was a risk factor of being bullied and bullying others. Additionally, long hours of online games were a risk factor of being bullied and bullying others, which was consistent with previous findings [27]. Meanwhile, online games with violence, confrontation, and other factors that easily cause conflict were also a risk factor for perpetration and victimization, which was also consistent with some previous findings [48]. Games with violence can lead to an increase in aggressive behavior, aggressive cognition, aggressive emotion and physical arousal [49]. In addition, games with violence will affect users’ hostile expectations. Bushman’s study [50] found that bloody images in games with violence can induce higher levels of stateful hostility. As shown in Table 2, Table 4 and Table 5, Anxiety symptom scores were associated with cyberbullying in social media, but not associated with cyberbullying in online games, which may be because online games are a form of entertainment that can relieve daily life stress. Internet addiction was a risk factor of cyberbullying in online games, which may be because students spend more time on games [22]. Therefore, those who spend more time on online games were more likely to be bullied online. However, Internet addiction has nothing to do with bullying others in online games. Moreover, online games attract players through unique game ways, making many game players immersed in games. Although they may be bullied by others, they do not care too much about bullying, they do not fight back and they do not take the initiative to bully others. They only want to satisfy their entertainment needs in the game world. Therefore, although they may be bullied by others, they will not bully others because they think bullying is meaningless behavior. Grade level, parents’ marital status, whether a local or single child family were not related to cyberbullying, as personal information was not normally disclosed on the Internet. Additionally, even if they see ethnic discrimination, regional discrimination, etc., they generally do not think it is against themselves. During the pre-survey, a student said in the interview that if it was some positive news about his hometown, the comment area would be relatively normal. However, if it was some negative news, the comment area would start to conduct “Regional Discrimination”, such as denying people of the entire region. He also said “I do not like being labeled and to be represented. There was bad news in every province, but why not criticize the event itself and attack the people of the entire province instead? It was a terrible feeling”. This also indicated that there were differences between cyberbullying and traditional bullying, and that these risk factors in traditional bullying studies cannot be applied to cyberbullying.

The above study result provided insights that targeted and effective measures should be taken to prevent college students from cyberbullying. Professional psychological counseling teachers need to timely provide psychological assistance online and offline for college students in need to reduce their anxiety and keep them mentally healthy. Besides, school administrators and parents should teach college students how to use the Internet reasonably and suggest that they should control the length of time that they surf the Internet every day. In addition, for college students with an Internet addiction disorder, professional psychological experts need to carry out effective intervention measures to reduce the risk of their involvement in cyberbullying. Finally, college students should limit their exposure to violent online games, because it will increase the risk of involvement in cyberbullying.

### 4.3. Strength and Limitation

Compared with other studies, this study did not copy the questionnaire that has been proved effective in other countries but not suitable for China. Instead, after reviewing a lot of literature, conducting pre-survey and interviews, we determined a suitable cyber-bullying questionnaire for China. At the same time, problems unsuitable for domestic situations, such as e-mail bullying and text bullying, were removed to make the questionnaire more authentic and effective. In addition, we put forward some targeted preventive measures according to the research results

Of course, this study also has its limitations. Firstly, this study was a cross-sectional study, which was hard to prove a causal relationship. Secondly, online questionnaires were difficult to control, which affects the accuracy of the answer. Thirdly, respondents would exaggerate or conceal the fact, thus forming a socially favorable response when they were asked some sensitive questions. Our team group is conducting a prospective design to avoid the above limitations, whose results will be reported in the future. Finally, our study treated cyberbullying as a homogeneous problem without identifying and segmenting according to the different kinds of cyberbullying. As the purpose of our study was to understand the incidence of cyberbullying in online games and social media among Chinese college students and its influencing factors, as well as the different characteristics of the occurrence of cyberbullying in these two channels. Future research can take this into account and further enrich the research content.

## 5. Conclusions

This study provided a comprehensive understanding of the incidence of cyberbullying in social media and online games and its associated factors among college students in China through a cross-sectional study. Moreover, 64.32% college students reported that they had suffered from cyberbullying, and 25.98% reported bullying others online during the semester in this study. We have identified several associated factors of cyberbullying in social media and online games through multivariate logistic regression analysis, including gender, anxiety symptoms, Internet addiction, game time, and violent elements in games. In conclusion, cyberbullying in social media and online games was prevalent among college students in China. The above data provided insights that targeted and effective measures should be taken to prevent college students from cyberbullying. The nature of the cross-sectional study prevents us from making a causal inference. Besides, our data is self-reported, recall and report bias would be unavoidable. Thus, future studies should conduct prospective studies to further prove the impact of these risk factors on cyberbullying. What is more, in further studies, the aim should be a wider sample, carrying out several data collection about the same subjects, and the combination of the use of self-reports and hetero-reports, which could make the results more reliable. This study also presents the prevalence of cyberbullying among Chinese college students and its associated factors for other researchers in the field of cyberbullying, which can provide a scientific reference for the design and implementation of future intervention studies on the prevention of cyberbullying among college students. At the same time, the results of this study also provide a scientific basis for policy formulation to prevent college students from cyberbullying. Therefore, policymakers and school administrators are supposed to take these associated factors of cyberbullying into full consideration when formulating policies to prevent cyberbullying among college students.

## Figures and Tables

**Table 1:** Univariate analysis of cyberbullying in social media among college students.

Characteristics	ALL N = 897	Victim N = 461			Perpetrator N = 174		
	*n* (%)	*n* (%)	*χ²*	*p*-Value	*n* (%)	*χ*²	*p*-Value
Gender			52.909	<0.001		50.525	<0.001
Male	393 (43.8)	256 (65.14)			118 (30.03)		
Female	504 (56.2)	205 (40.67)			56 (11.11)		
Daily Online hours			7.475	0.113		7.901	0.095
1–4	199 (22.1)	86 (43.21)			29 (14.57)		
4–7	431 (48.1)	236 (54.76)			81 (18.79)		
7–10	147 (16.4)	77 (52.38)			36 (24.48)		
10–13	85 (9.4)	43 (50.59)			22 (25.88)		
>13	35 (3.8)	19 (54.26)			6 (17.14)		
College			3.445	0.074		6.983	0.013
Medical college	746 (83.2)	373 (50.00)			133 (17.83)		
Others	151 (16.8)	88 (58.29)			41 (27.15)		
Grade			5.736	0.220		2.014	0.733
1	283 (31.5)	154 (54.41)			51 (18.02)		
2	211 (23.5)	95 (45.02)			40 (18.96)		
3	220 (24.5)	111 (50.45)			45 (20.45)		
4	171 (19.1)	95 (55.56)			37 (21.64)		
5	12 (1.3)	6 (50.00)			1 (8.33)		
Nationality			0.032	0.859		0.136	0.712
Han	884 (98.6)	454 (51.36)			172 (19.46)		
Others	13 (1.4)	7 (53.85)			2 (15.38)		
Only child family or not			2.559	0.110		0.926	0.336
Yes	242 (27.0)	135 (55.79)			52 (21.49)		
No	655 (73.0)	326 (49.77)			122 (18.63)		
Natives or not			0.173	0.678		0.052	0.819
Yes	717 (79.9)	366 (51.04)			138 (19.25)		
No	180 (20.1)	95 (52.78)			36 (20.00)		
Marital status of parents			1.895	0.755		2.936	0.569
Original married	818 (91.2)	416 (50.86)			161 (19.68)		
Widowed	21 (2.3)	11 (52.38)			4 (19.08)		
Divorced	25 (2.8)	16 (64.00)			4 (16.00)		
Remarried	28 (3.1)	15 (53.57)			3 (10.71)		
Others	5 (0.6)	3 (60.00)			2 (40.00)		

**Table 2:** Univariate analysis of cyberbullying in social media and self-esteem, anxiety and Internet addiction among college students.

Scale Score	Victim				Perpetrator			
	Yes *X ± SD*	No *X ± SD*	*t*	*p*-Value	Yes *X ± SD*	No *X ± SD*	*t*	*p*-Value
Self-esteem score	30.13 ± 4.81	30.42 ± 4.59	−0.0935	0.350	30.18 ± 5.16	30.30 ± 4.60	−0.296	0.767
Anxiety self-rating score	42.04 ± 10.51	38.00 ± 7.53	6.647	<0.001	43.74 ± 11.86	36.70 ± 8.48	4.764	<0.001
Internet addiction score	44.29 ± 10.31	41.27 ± 10.01	4.442	<0.001	45.56 ± 10.68	42.16 ± 10.07	3.949	<0.001

**Table 3:** Univariate analysis of cyberbullying in online games among college students.

Characteristics	ALL N = 600 n%	Victim N = 355			Perpetrator N = 122		
		*n* (%)	*χ²*	*p*-Value	*n* (%)	*χ*²	*p*-Value
Gender			33.658	<0.001		56.603	<0.001
Male	330 (55.0)	230 (69.70)			104 (31.52)		
Female	270 (45.0)	125 (46.30)			18 (6.66)		
Game time			37.996	<0.001		45.184	<0.001
<7 h	368 (61.3)	182 (49.46) ^a^			45 (12.77) ^a^		
7–14 h	153 (25.5)	113 (73.86) ^b^			47 (30.71) ^b^		
14–21 h	42 (7.0)	30 (71.43) ^b^			12 (28.57) ^b^		
>21 h	37 (6.2)	30 (81.08) ^b^			18 (48.64) ^b^		
College			2.156	0.170		0.127	0.721
Medical college	485 (80.8)	280 (57.73)			100 (20.62)		
Others	115 (19.2)	75 (65.22)			22 (19.13)		
Grade			3.804	0.433		2.230	0.694
1	195 (32.5)	116 (59.49)			36 (18.46)		
2	129 (21.5)	80 (62.02)			26 (20.63)		
3	153 (25.5)	86 (56.21)			37 (24.18)		
4	115 (19.2)	66 (57.39)			22 (19.13)		
5	8 (1.3)	7 (87.50)			1 (12.50)		
Nationality			1.310	0.252		0.953	0.329
Han	591 (98.5)	348 (58.88)			119 (20.14)		
Others	9 (1.5)	7 (77.78)			3 (33.33)		
Only child or not			0.498	0.481		0.139	0.245
Yes	176 (29.3)	108 (61.36)			41 (23.29)		
No	424 (70.7)	247 (58.25)			81 (19.10)		
Natives or not			0.386	0.534		0.715	0.398
Yes	470 (78.3)	277 (58.93)			99 (21.06)		
No	130 (21.7)	80 (61.54)			23 (17.69)		
Marital status of parents			8.799	0.066		5.233	0.265
Original married	548 (91.3)	334 (60.95)			117 (21.35)		
Widowed	13 (2.2)	6 (46.15)			2 (15.38)		
Divorced	16 (2.7)	6 (37.50)			1 (6.25)		
Remarried	19 (3.2)	7 (36.84)			1 (5.26)		
Others	4 (0.7)	2 (50.00)			1 (25.00)		
Violent element in game or nor			107.258	<0.001		40.730	<0.001
Yes	349 (58.2)	268 (76.79)			102 (29.23)		
No	251 (41.8)	87 (34.66)			20 (7.97)		

a, b: Different letter represents a statistically significant difference in rates between the two groups.

**Table 4:** Univariate analysis of cyberbullying in online game and self-esteem, anxiety and Internet addiction among college students.

Scale Score	Victim				Perpetrator			
	Yes *X ± SD*	No *X ± SD*	*t*	*p*-Value	Yes *X ± SD*	No *X ± SD*	*t*	*p*-Value
Self-esteem score	30.37 ± 4.89	30.51 ± 4.57	−0.347	0.729	30.52 ± 4.84	30.40 ± 4.74	0.259	0.796
Anxiety self-rating score	40.38 ± 9.72	39.34 ± 8.83	1.355	0.182	41.01 ± 10.51	39.69 ± 9.05	1.398	0.163
Internet addiction score	43.59 ± 10.55	41.92 ± 9.46	1.991	0.047	43.28 ± 12.04	42.81 ± 9.61	0.396	0.693

**Table 5:** Multivariate logistic regression analysis on associated factors of cyberbullying among College Students.

Characteristics	*β*	S. E	*p*-Value	*Adjusted OR*	95%*CI*
The victim in social media					
Male (reference: female)	1.161	0.148	<0.001	3.192	2.389–4.264
Anxiety self-rating score	0.047	0.009	<0.001	1.048	1.030–1.066
Internet addiction score	0.025	0.008	0.001	1.025	1.010–1.040
The perpetrator in social media					
Male (reference: female)	1.392	0.188	<0.001	4.024	2.738–5.817
Anxiety self-rating score	0.043	0.010	<0.001	1.048	1.030–1.066
Internet addiction score	0.029	0.010	0.003	1.029	1.010–1.049
The victim in the online game					
Male (reference: female)	0.761	0.204	<0.001	2.141	1.437–3.192
Violent element in game	1.735	0.189	<0.001	5.668	3.911–8.214
Game time	0.339	0.129	0.008	1.404	1.091–1.807
Internet addiction score	0.018	0.010	0.046	1.018	1.001–1.037
The perpetrator in the online game					
Male (reference: female)	1.542	0.285	<0.001	4.676	2.673–8.179
Violent element in game	1.379	0.271	<0.001	3.969	2.332–6.756
Game time	0.379	0.120	0.002	1.461	1.155–1.847

## Data Availability

The datasets used and analyzed in the study are available from the corresponding author on reasonable request.

## Supplementary Materials

The following are available online at https://www.mdpi.com/article/10.3390/ijerph18094819/s1, Table S1: This scale is used to understand your situation of surfing online. Please read the following sentences carefully and choose the option that best suits your situation.

## References

[1] Kowalski R.M., Limber S.P., Agatston P.W. (2008). Cyber bullying: Bullying in the digital age. J. Adolesc. Health.

[2] Willard N.E. (2007). Cyberbullying and Cyberthreats: Responding to the Challenge of Online Social Aggression, Threats, and Distress.

[3] Beran T.N., Rinaldi C., Bickham D.S., Rich M. (2012). Evidence for the need to support adolescents dealing with harassment and cyber-harassment: Prevalence, progression, and impact. Sch. Psychol. Int..

[4] Jenaro C., Flores N., Frías C.P. (2017). Systematic review of empirical studies on cyberbullying in adults: What we know and what we should investigate. Aggress. Violent Behav..

[5] Hinduja S., Patchin J.W. (2008). Cyberbullying: An Exploratory Analysis of Factors Related to Offending and Victimization. Deviant Behav..

[6] Bonanno R.A., Hymel S. (2013). Cyber Bullying and Internalizing Difficulties: Above and Beyond the Impact of Traditional Forms of Bullying. J. Youth Adolesc..

[7] Khine A.T., Saw Y.M., Htut Z.Y., Khaing C.T., Soe H.Z., Swe K.K., Thike T., Htet H., Saw T.N., Cho S.M. (2020). Assessing risk factors and impact of cyberbullying victimization among university students in Myanmar: A cross-sectional study. PLoS ONE.

[8] Peng Z., Klomek A.B., Li L., Su X., Sillanmäki L., Chudal R., Sourander A. (2019). Associations between Chinese adolescents subjected to traditional and cyber bullying and suicidal ideation, self-harm and suicide attempts. BMC Psychiatry.

[9] Ojanen T.T., Boonmongkon P., Samakkeekarom R., Samoh N., Guadamuz T.E. (2015). Connections between online harassment and offline violence among youth in Central Thailand. Child Abus. Negl..

[10] Katzer C., Fetchenhauer D., Belschak F. (2009). Cyberbullying: Who Are the Victims?. J. Media Psychol. Theor. Methods Appl..

[11] Alhabash S., Mcalister A.R., Hagerstrom A., Quilliam E.T., Rifon N.J., Richards J.I. (2013). Between likes and shares: Effects of emotional appeal and virality on the persuasiveness of anticyberbullying messages on Facebook. Cyberpsychol. Behav. Soc. Netw..

[12] Aricak O.T. (2009). Psychiatric Symptomatology as a Predictor of Cyberbullying among University Students. Eurasian J. Educ. Res..

[13] Doane A.N., Boothe L.G., Pearson M.R., Kelley M.L. (2016). Risky electronic communication behaviors and cyberbullying victimization: An application of Protection Motivation Theory. Comput. Hum. Behav..

[14] Elipe P., Mora-Merchán J.A., Ortega-Ruiz R., Casas J.A. (2015). Perceived emotional intelligence as a moderator variable between cybervictimization and its emotional impact. Front. Psychol..

[15] Feinstein B.A., Bhatia V., Davila J. (2014). Rumination Mediates the Association between Cyber-Victimization and Depressive Symptoms. J. Interpers. Violence.

[16] Francisco S.M., Simao A.M.V., Ferreira P.C., Martins M.J.D.D. (2015). Cyberbullying: The hidden side of college students. Comput. Hum. Behav..

[17] Gahagan K., Vaterlaus J.M., Frost L.R. (2016). College student cyberbullying on social networking sites. Comput. Hum. Behav..

[18] Johnston P., Tankersley M., Joenson T., Hupp M., Buckley J., Redmond-Mcgowan M., Zanzinger A., Poirier A., Walsh A. (2014). Motivations Behind “Bullies then Offenders” Versus “Pure Bullies”: Further suggestions for Anti-Bully Education and Practice. Education.

[19] Selkie E.M., Kota R., Chan Y.F., Moreno M. (2015). Cyberbullying, depression, and problem alcohol use in female college students: A multisite study. Cyberpsychol. Behav. Soc. Netw..

[20] Tosun N. (2016). Cyberbully and Victim Experiences of Pre-Service Teachers. Eur. J. Contemp. Educ..

[21] Coelho V.A., Romão A.M. (2018). The relation between social anxiety, social withdrawal and (cyber)bullying roles: A multilevel analysis. Comput. Hum. Behav..

[22] Smith P.K., Mahdavi J., Carvalho M., Fisher S., Russell S., Tippett N. (2010). Cyberbullying: Its nature and impact in secondary school pupils. J. Child Psychol. Psychiatry.

[23] Zhang S., Zhang Y., Yuan B. (2019). Mediating effect of self-esteem and empathy on the relationship between loneliness and cyber-bulling in middle and high school students in Liaoning Province. Wei Sheng Yan Jiu J. Hyg. Res..

[24] Rosenberg M. (1965). Self Esteem and the Adolescent. (Economics and the Social Sciences: Society and the Adolescent Self-Image). N. Engl. Q..

[25] Craske M.G., Stein M.B. (2016). Anxiety. Lancet.

[26] Block J.J. (2008). Issues for DSM-V: Internet addiction. Am. J. Psychiatry.

[27] Tang W.Y., Fox J. (2016). Men’s harassment behavior in online video games: Personality traits and game factors. Aggress. Behav..

[28] Erdur-Baker O., Kavsut F. (2007). Cyber bullying: A new face of peer bullying. Eurasian J. Educ. Res..

[29] Cretin B., Yaman E., Peker A. (2011). Cyber victim and bullying scale: A study of validity and reliability. Comput. Educ..

[30] Watts L.K., Wagner J., Velasquez B., Behrens P.I. (2017). Cyberbullying in higher education: A literature review. Comput. Hum. Behav..

[31] Van Hee C., Jacobs G., Emmery C., Desmet B., Lefever E., Verhoeven B., De Pauw G., Daelemans W., Hoste V. (2018). Automatic detection of cyberbullying in social media text. PLoS ONE.

[32] Byrne E., Vessey J.A., Pfeifer L. (2018). Cyberbullying and Social Media: Information and Interventions for School Nurses Working With Victims, Students, and Families. J. Sch. Nurs. Off. Publ. Natl. Assoc. Sch. Nurses.

[33] Cagirkan B., Bilek G. (2021). Cyberbullying among Turkish high school students. Scand. J. Psychol..

[34] Pieterse J. (1989). Society and the Adolescent Self-Image.

[35] William W.K.Z.M.D. (1971). A rating instrument for anxiety disorders. Psychosom.

[36] Bai Y., Fan F.M. (2005). Revision and application of college students’ network dependence measurement tools. Psychol. Dev. Educ..

[37] Nie N.H., Bent D.H., Hull C.H. (2003). SPSS Statistical Package for the Social Sciences. Encycl. Inf. Syst..

[38] Zhu H., Shi F., An L., Yin X., Fu M., Wang Y. (2016). Analysis on prevalence of cyberbullying in college students in China. J. Jilin Univ..

[39] (2015). The 35th Statistical Report on Internet Development in China.

[40] (2019). The 44th Statistical Report on Internet Development in China.

[41] Dennis E.A., Martin H.B., Anthony B., Shawn B. (1992). Logistic regression analysis. Med.mahidol.ac.th.

[42] Wickens, Thomas D. (1998). Categorical Data Analysis. Annu. Rev. Psychol..

[43] Walker C.M., Sockman B.R., Koehn S. (2011). An Exploratory Study of Cyberbullying with Undergraduate University Students. Techtrends Link. Res. Pract. Improv. Learn..

[44] Sminkey L. (2008). World report on child injury prevention. Inj. Prev..

[45] Campbell M.A., Slee P.T., Spears B., Butler D., Kift S. (2013). Do cyberbullies suffer too? Cyberbullies’ perceptions of the harm they cause to others and to their own mental health. Sch. Psychol. Int..

[46] Davis R.A. (2001). A cognitive-behavioral model of pathological Internet use. Comput. Hum. Behav..

[47] Arpaci I., Abdeljawad T., Baloğlu M., Kesici Ş., Mahariq I. (2020). Mediating Effect of Internet Addiction on the Relationship Between Individualism and Cyberbullying: Cross-Sectional Questionnaire Study. J. Med. Internet Res..

[48] Anderson C.A., Shibuya A., Ihori N., Swing E.L., Saleem M. (2010). Violent Video Game Effects on Aggression, Empathy, and Prosocial Behavior in Eastern and Western Countries: A Meta-Analytic Review. Psychol. Bull..

[49] Anderson C.A., Dill K.E. (2000). Video Games and Aggressive Thoughts, Feelings, and Behavior in the Laboratory and in Life. J. Personal. Soc. Psychol..

[50] Barlett C.P., Harris R.J., Bruey C. (2008). The effect of the amount of blood in a violent video game on aggression, hostility, and arousal. J. Exp. Soc. Psychol..

