# Supplementary material for: Cyberbullying in Social Media and Online Games among Chinese College Students and Its Associated Factors
Source: Int J Environ Res Public Health. 2021 Apr 30;18(9):4819. doi: 10.3390/ijerph18094819 (PMC8125715; doi:10.3390/ijerph18094819)
Supplement: Supplementary file 1 [file ijerph-18-04819-s001.zip › ijerph-1188600-supplementary.pdf]

This scale is used to understand your situation of surfing online. Please read the following sentences carefully and choose the option that best suits your situation.

	**strongly inconsistent**	**inconsistent**	**consistent**	**very consistent**
I have been told more than once that I spend too much time on the Internet	○	○	○	○
I find myself surfing the Internet longer and longer	○	○	○	○
No matter how tired I am, I feel energetic when I surf the Internet.	○	○	○	○
In fact, every time I just want to stay online for a while, but I often stay for a long time	○	○	○	○
I have slept less than four hours a day more than once because of the Internet	○	○	○	○
Since last semester, I have spent more time on the Internet every week than before	○	○	○	○
As long as I don't surf the Internet for a period of time, I will be depressed	○	○	○	○
I find myself so immersed in the Internet that I have less interaction with my friends	○	○	○	○
I used to have backache or other physical discomfort because of surfing the Internet	○	○	○	○
When I wake up every morning, the first thing I think of is surfing the Internet.	○	○	○	○
The Internet has caused some negative effects on my study or work	○	○	○	○

As long as I don't surf the Internet for a period of time, I feel like I've missed something	○	○	○	○
Because of the Internet, I usually have less leisure time	○	○	○	○
Every time I get off the Internet, I actually have to do something else, but I can't help surfing on the Internet again	○	○	○	○
Without the Internet, my life would be no fun	○	○	○	○
Surfing the Internet has a negative effect on my body	○	○	○	○
I used to sleep less so that I could spend more time on the Internet	○	○	○	○
I have to spend more time online to be satisfied than I used to	○	○	○	○
I was out of spirits during the day because I stayed up late surfing the Internet	○	○	○	○
